# Burden of Secondary Hyperparathyroidism Among Patients on Hemodialysis: A Cross-Sectional Study

**DOI:** 10.7759/cureus.98534

**Published:** 2025-12-05

**Authors:** Muhammad Ikram, Hayat Ullah, Muhammad Ayaz, Arbab Muhammad Ali, Khushhal Khan Nungyaal, Shad Muhammad, Waqas Ur Rahman

**Affiliations:** 1 Department of Nephrology, Lady Reading Hospital Medical Teaching Institution, Peshawar, PAK; 2 Department of Pathology, Khyber Medical College, Peshawar, PAK

**Keywords:** dialysis duration, end-stage renal disease, hemodialysis, mineral bone disorder, secondary hyperparathyroidism

## Abstract

Background: Secondary hyperparathyroidism (SHPT) is a frequent complication in patients with end-stage renal disease (ESRD) undergoing hemodialysis. It results from imbalances in calcium, phosphate, and vitamin D metabolism due to impaired renal function.

Objective: This study aimed to determine the frequency of SHPT among hemodialysis-dependent ESRD patients in a tertiary care hospital in Khyber Pakhtunkhwa.

Methods: A cross-sectional study was conducted in the Nephrology Department of Lady Reading Hospital, Peshawar, from August 2023 to January 2024. A total of 127 patients between 18 and 75 years of age, undergoing maintenance hemodialysis for at least six months, were included. SHPT was defined in accordance with the Kidney Disease: Improving Global Outcomes (KDIGO) 2017 guidelines as an intact parathyroid hormone (iPTH) level greater than nine times the upper reference limit in hemodialysis patients. Data were analyzed using IBM SPSS Statistics for Windows, version 25, with stratification based on age, dialysis duration, diabetes status, and hypertension.

Results: Out of 127 patients, 25 (19.7%) were found to have SHPT. The mean age of the study population was 51.8 ± 15 years, with 77 (60.6%) males and 50 (39.4%) females. Diabetes was present in 70 (55.1%) patients, while 83 (65.4%) were hypertensive. SHPT was observed more frequently among male patients, those with a longer duration of dialysis, and individuals with coexisting diabetes and hypertension.

Conclusion: SHPT was identified in nearly one-fifth of hemodialysis patients, with higher prevalence among males, diabetics, hypertensives, and those on prolonged dialysis. These findings highlight the importance of regular biochemical monitoring and early intervention to reduce SHPT-related complications and improve long-term outcomes in hemodialysis-dependent patients.

## Introduction

Secondary hyperparathyroidism (SHPT) is a frequent and serious complication among patients with end-stage renal disease (ESRD) on chronic hemodialysis. It results from impaired regulation of calcium, phosphorus, and vitamin D metabolism, leading to excessive secretion of parathyroid hormone (PTH) and progressive parathyroid gland hyperplasia [1,2]. The condition not only affects mineral balance but also contributes to skeletal abnormalities, vascular calcification, cardiovascular events, and overall increased morbidity and mortality [3,4].

The burden of SHPT is particularly significant in hemodialysis-dependent patients, where altered bone metabolism leads to renal osteodystrophy, fractures, and reduced quality of life [5]. Despite advances in therapy, including phosphate binders, vitamin D analogs, calcimimetics, and surgical interventions, optimal control of SHPT remains difficult in routine clinical practice [6,7]. Poor control is associated with both short- and long-term adverse outcomes, underscoring the importance of evaluating its prevalence and determinants in dialysis populations [8].

Large multicenter studies, such as those conducted by the Dialysis Outcomes and Practice Patterns Study (DOPPS), have reported wide variations in SHPT prevalence across countries and dialysis units, reflecting differences in treatment strategies, dialysis adequacy, and availability of medications [9]. However, such aggregated data often mask local disparities in disease burden, particularly in regions where resources are limited or where guideline-directed therapies are not consistently applied [10]. Single-center investigations are therefore essential for identifying center-specific challenges and guiding targeted interventions [11].

Moreover, the clinical consequences of uncontrolled SHPT extend beyond bone health. Elevated PTH and calcium-phosphate product levels have been linked to vascular calcification, left ventricular hypertrophy, and higher mortality in dialysis patients [12, 13]. The interplay between SHPT and other components of chronic kidney disease-mineral and bone disorder (CKD-MBD) further complicates management, requiring individualized assessment and monitoring [14].

Given these considerations, there is a clear need to gain clearer insight into the local burden of SHPT in hemodialysis populations. Evaluating its prevalence, severity, and associated biochemical and clinical parameters in a single-center setting can provide valuable insights into practice gaps and inform strategies for improved patient care. This study, therefore, seeks to characterize the burden of SHPT in hemodialysis-dependent patients at our center, with the broader aim of contributing evidence that may help optimize management strategies and improve outcomes in similar clinical settings.

## Materials and methods

This cross-sectional study was conducted in the hemodialysis unit of MTI Lady Reading Hospital, Peshawar, a major tertiary care center in Khyber Pakhtunkhwa, Pakistan, over a six-month period from August 2023 to January 2024. Adult patients aged 18 years or above with ESRD who had been on regular maintenance hemodialysis for at least six months were considered eligible. Patients with acute kidney injury, prior parathyroidectomy, chronic liver disease, active malignancy, or pregnancy were excluded to avoid confounding factors that could influence mineral and bone metabolism.

The minimum required sample size was calculated using the OpenEpi calculator, assuming a 30% prevalence of secondary hyperparathyroidism (SHPT) in ESRD patients, with a 95% confidence level and an 8% margin of error, resulting in a sample size of 127 participants [14]. A non-probability consecutive sampling technique was employed, whereby all eligible patients presenting during the study period were enrolled. Ethical approval was obtained from the Institutional Review and Ethics Board (IRB) of MTI, Lady Reading Hospital, Peshawar (Reference No. 823/LRH/MTI, dated 17 July 2023). Written informed consent was taken from all participants prior to enrollment.

Data collection was carried out using a structured proforma, which included demographic characteristics, dialysis-related details, comorbidities, and medications such as phosphate binders, vitamin D preparations, and calcimimetics. Pre-dialysis venous blood samples were obtained during mid-week dialysis sessions to minimize intra-dialytic variability. Laboratory investigations included measurements of intact parathyroid hormone (iPTH), calcium, and phosphate, all performed in the hospital’s central laboratory. SHPT was defined according to the KDIGO 2017 CKD-MBD guidelines as an iPTH level greater than nine times the upper reference limit in hemodialysis patients [15].

The primary outcome of interest was the prevalence of SHPT among hemodialysis patients. Secondary outcomes included the association of SHPT with age, sex, comorbidities, and dialysis duration and frequency. Data analysis was performed using IBM SPSS Statistics for Windows, version 26.0 (released 2018, IBM Corp., Armonk, NY). Descriptive statistics were reported as mean ± standard deviation for continuous variables and frequency with percentage for categorical variables. The Chi-square test was applied to assess associations between categorical variables, and a p-value of ≤0.05 was considered statistically significant.

## Results

The study included 127 patients with ESRD receiving maintenance hemodialysis at MTI Lady Reading Hospital. Of these, 77 (60.6%) were male, and 50 (39.4%) were female. The mean age of participants was 51.8 ± 15.0 years. Comprehensive baseline demographic characteristics are summarized in Table 1.

**Table 1:** Descriptive statistics of the study participants iPTH: intact parathyroid hormone

	N	Minimum	Maximum	Mean	Std. deviation
Age (years)	127	18	73	51.85	15.059
Duration of hemodialysis (months)	127	11	45	21	10.28
Serum calcium (mg/dl)	127	6.2	9.2	7.774	.5527
Serum phosphorus (mg/dl)	127	2.9	10.0	6.683	1.9934
Serum iPTH (pg/ml)	127	13	990	370.06	280.211

Among the study participants, 70 (55.1%) were diabetic, and 83 (65.4%) were hypertensive. With respect to dialysis frequency, eight patients (6.3%) underwent hemodialysis once weekly, while the majority, 119 patients (93.7%), were maintained on twice-weekly sessions. Based on dialysis duration, the participants were divided into three categories: 13 patients (10.2%) had been receiving hemodialysis for six to 12 months, 76 patients (59.8%) for 12-24 months, and 38 patients (29.9%) for more than 24 months. Furthermore, when stratified according to serum iPTH levels, patients were classified into three groups, i.e., low, target range, and high, as summarized in Table 2.

**Table 2:** Distribution of serum iPTH levels among the study population iPTH: intact parathyroid hormone

PTH category		Frequency	Percent	Valid percent	Cumulative percent
Valid	Low	28	22.0	22.0	22.0
	Target	74	58.3	58.3	80.3
	High	25	19.7	19.7	100.0
	Total	127	100.0	100.0	

As illustrated in Figure 1, SHPT was identified in 19.6% of the study population. While SHPT was observed more frequently in males than in females, the difference did not reach statistical significance (p = 0.13). A significant association was observed with dialysis duration, as patients undergoing hemodialysis for more than 24 months had markedly higher iPTH levels compared to those with a shorter treatment history (p = 0.012), as shown in Table 3. Interestingly, SHPT was found to be more prevalent among non-diabetic (p = 0.012) and non-hypertensive patients (p = 0.003).

**Table 3:** Distribution of serum PTH in relation to hemodialysis duration PTH: parathyroid hormone

		Duration of hemodialysis (months)			Total
		6-12 months	12-24 months	>24 months	
PTH category	Low	5	19	4	28
	Target	7	47	20	74
	High	1	10	14	25
Total		13	76	38	127

## Discussion

In our study, SHPT was identified in 19.6% of patients undergoing maintenance hemodialysis. This proportion is lower than the prevalence reported in many international studies, where SHPT rates vary between 30% and 70%, largely influenced by patient characteristics and treatment practices [16,17]. For instance, Rahimian et al. documented a prevalence of 45% among Iranian hemodialysis patients, with significant associations to both biochemical and radiological markers [18]. On the other hand, our figure is higher than that reported from Sheikh Zayed Hospital, Lahore, where SHPT was found in only 10% of dialysis patients [19]. Such differences emphasize the impact of regional disparities in dialysis adequacy, access to medications, and nutritional care.

We also observed a clear link between longer dialysis duration and higher SHPT prevalence. This supports the established pathophysiology, whereby prolonged exposure to hyperphosphatemia, reduced calcitriol synthesis, and parathyroid hyperplasia drive persistent elevations in PTH [20]. Jean et al. similarly showed in a 36-month follow-up of incident dialysis patients that severe SHPT developed in 18%, particularly in those with high baseline PTH and CTX, underlining the value of early biochemical surveillance [21]. Collectively, these findings highlight the need for routine monitoring and timely preventive measures.

An unexpected observation in our cohort was the higher prevalence of SHPT among non-diabetic and non-hypertensive patients. Although counterintuitive, Jean et al. also noted lower SHPT rates in diabetics, possibly reflecting competing mortality risks or altered bone metabolism [21]. Survival bias may therefore explain why patients with fewer comorbidities appear more likely to manifest SHPT over time. Further prospective work is required to clarify this association.

We found no significant gender difference in SHPT prevalence, consistent with earlier reports indicating that sex alone does not independently influence PTH levels [16,22]. Nonetheless, the tendency toward higher prevalence in males may reflect subtle variations in bone turnover or treatment compliance.

The clinical implications of these findings are important. SHPT remains a driver of renal osteodystrophy, skeletal fractures, vascular calcification, and cardiovascular complications [17, 20]. KDIGO guidelines strongly recommend regular assessment of PTH, calcium, and phosphate for early intervention [15]. In resource-limited settings, however, challenges such as cost and restricted access to calcimimetics or vitamin D analogs demand practical alternatives, including dietary counseling and affordable phosphate binders.

This was a single-center study with a modest sample size; the findings may have limited generalizability to broader populations. Moreover, the cross-sectional design restricts the ability to infer temporal or causal relationships between risk factors and SHPT. In addition, resource constraints prevented the inclusion of other relevant biochemical markers, such as vitamin D levels, alkaline phosphatase, which might have provided a more comprehensive assessment of CKD-MBD. Despite these limitations, the study highlights important trends and underscores the need for larger, multicenter studies in similar low-resource settings.

## Conclusions

Our study highlights that SHPT continues to be a meaningful challenge for patients on long-term hemodialysis in our setting, affecting nearly one in five individuals. The condition was seen more often in those with prolonged dialysis duration, while patterns across diabetes, hypertension, and gender suggest a complex relationship between comorbidities and disease expression. These findings reinforce the importance of regular monitoring of calcium, phosphate, and PTH levels so that timely measures can be taken to reduce the risk of bone disease, cardiovascular complications, and poor quality of life in this vulnerable group.

Although limited to a single center with a modest sample size, this work adds useful local evidence where data are lacking. Broader studies are needed to confirm these trends and guide policies that improve care in similar low-resource settings. Strengthening access to affordable therapies, encouraging early detection, and focusing on patient education may help reduce the burden of SHPT and ultimately improve survival and well-being in dialysis patients.
